# Prebiotics Inulin Metabolism by Lactic Acid Bacteria From Young Rabbits

**DOI:** 10.3389/fvets.2021.719927

**Published:** 2021-10-01

**Authors:** Yuan-ting Zhu, Shuang-ming Yue, Rui-tong Li, Shi-xiu Qiu, Zhen-Ying Xu, Yi Wu, Jin Yao, Yong Zuo, Ke-juan Li, Yang Li

**Affiliations:** ^1^College of Life Science, Sichuan Normal University, Chengdu, China; ^2^Department of Bioengineering, Sichuan Water Conservancy College, Chengdu, China; ^3^Chengdu Academy of Agriculture and Forestry Sciences, Chengdu, China

**Keywords:** inulin, prebiotic, rabbit, *lacticaseibacillus paracasei*, synbiotic

## Abstract

Inulin as a commercial prebiotic could selectively promote the growth of beneficial gut microbes such as lactic acid bacteria (LAB). Whether LAB in rabbit gut possesses the capability to metabolize and utilize inulin is little known. Therefore, this study recovered 94 LAB strains from neonate rabbits and found that only 29% (28/94) could metabolize inulin with both species- and strain-specificity. The most vigorous inulin-degrading strain, *Lacticaseibacillus paracasei* YT170, could efficiently utilize both short-chain and long-chain components through thin-layer chromatography analysis. From genomic analysis, a predicted *fosRABCDXE* operon encoding putative cell wall-anchored fructan β-fructosidase, five fructose-transporting proteins and a *pts1BCA* operon encoding putative β-fructofuranosidase and sucrose-specific IIBCA components were linked to long-chain and short-chain inulin utilization respectively. This study provides a mechanistic rationale for effect of inulin administration on rabbits and lays a foundation for synbiotic applications aimed at modulating the intestinal microbiota of young rabbits.

## Introduction

Rabbits are important small herbivorous mammals widely raised for fur and meat production throughout the world for much of history. China is the largest rabbit meat producer in the world, followed by Europe (1). However, rabbits easily suffer from gastrointestinal diseases, especially in the weaning period, due to the fragility of their digestive system. According to the epidemic data and statistics in the rabbit industry, more than 75% of rabbits suffer from diarrhea, with an average mortality rate of 24% (2). Thus, since the 1950s antibiotics have been added to fodder, known as antibiotic growth promoters (AGPs) (3). While thesekept the disease under control for a short time, ultimately the use of antibiotics can easily cause significant changes in cecal flora, thus not only increasing digestive disorders and mortality in growing rabbits but also leading to eventual antibiotic residues in meat, with negative effects on food safety and further triggering drug resistance harmful to human health and the environment. Therefore, the European Union (EU) banned all AGPs in 2006, and China also banned the non-therapeutic use of the antibiotics in 2020. Therefore, more attention has been paid to researching safe and effective antibiotic alternatives in recent years.

The digestive tract disease which rabbits suffer from is often related to the imbalance of intestinal microorganisms, while healthy gut microbiota or their derived metabolites such as SCFAs contributes to the maturation of the gut barrier at the suckling-to-weaning transition (4) thus preventing and reducing the incidence of rabbit intestinal diseases, and had critical impacts on growth performance of rabbits (5). Therefore, it is extremely important to establish a healthy and stable intestinal flora (6). Probiotics, prebiotics or their combination, synbiotics, capable of modulating intestinal microbiota and further lowering the incidence of diarrhea, are important alternatives to antibiotic therapy in the livestock industry. To date, the research on alternatives to AGPs is limited in rabbits compared to other farm species (7).

Prebiotics usually refer to oligosaccharides that are not digested by animal enzymes, but can selectively stimulate certain intestinal bacterial species (8, 9); for instance, lactic acid bacteria (LAB) as important gut commensals could metabolize dietary carbohydrate as a carbon source without being digested in the upper gastrointestinal tract to obtain energy, thus promoting bacterial establishment and survival in the gut and enrichment of beneficial metabolites such as short-chain fatty acids (SCFAs), and as a consequence contributing to host health. The main commercial oligosaccharides at present are the fructo-oligosaccharides (FOS), inulin, galacto-oligosaccharides (GOS), and xylooligosaccharide (XOS) (10), etc. Among them, inulin is an important dietary fiber present in about 45,000 plant species as a major storage carbohydrate, particularly in *Helianthus tuberosus* (Jerusalem artichoke), *Dahlia pinnata* (dahlia), and *Cichorium intybus* (chicory). Inulin is a linear fructan composed of fructosyl units [beta-() linkage] and usually contains one terminal glucose moiety [alpha (1, 2) linkage] per molecule. These glycosidic linkages present in inulin make inulin resistant to hydrolysis by animal or human gastrointestinal enzymes. Therefore, inulin has been regarded as a common prebiotic (11) that could selectively promote the growth of gut beneficial microbes such as lactic acid bacteria. In rabbit feeding, inulin has been proven useful as a natural additive in antibiotic-free rabbit diets; for instance, it is routinely added as an ingredient in commercial feed for pet rabbits (12). In addition, oligofructose is routinely added at the levels of 1–3 g/kg in European feeds to improve gut health and reduce the mortality of rabbits. Also, prebiotic inulin is found to exhibit desirable changes in the gut of rabbits for augmenting gut health and growth performance in several studies (13, 14). The effect of inulin on gut health is associated with the capability of gut beneficial microbes to utilize inulin, which results in the selective growth of these microbes, the decline of the gastrointestinal pH, and production of short-chain fatty acids, etc. However, there is limited knowledge on the capacity of the resident bacteria, particularly lactic acid bacteria in rabbit gut, to consume inulin. Therefore, the present study recovered pure lactic acid bacteria strains from rabbit fecal samples and examined their inulin-utilizing capability, which provides a mechanistic rationale for the effect of inulin administration on rabbits. Furthermore, the most vigorous inulin-degrading strain was subjected to the investigation of metabolic characteristics and genomic sequencing and analysis for mining gene clusters related to inulin metabolism, which laid a foundation for designing a synbiotic aimed at modulating the gut microbiota in the early life of rabbits.

## Materials and Methods

### Materials and Reagents

Inulin (CAS No. 9005-80-5) was purchased from Sigma-Aldrich (Steinheim, Germany). Glass plates pre-coated with silica gel GF_254_ (Qingdao Haiyang Chemical Co., Ltd., Qingdao, China) were used for thin-layer chromatography (TLC) analysis. The Wizard Genomic DNA Purification kit (Promega Italia S.r.l., Milan, Italy) was employed for genomic DNA extraction from isolated pure strains. Universal primers 27F 5′-AGAGTTTGATCCTGGCTCAG-3′ and 1492R 5′-GGTTACCTTGTTACGACTT-3′ and a commercial 2× Taq PCR Master Mix were synthesized and provided by Shanghai Sangon Biotechnology Co., Ltd. (Shanghai, China) for PCR amplification. All other reagents used in this work were of analytical grade or higher.

### Culture Media

LAMVAB medium with vancomycin with a final concentration of 20 mg/L was used to isolate lactobacilli in feces, which was prepared following published protocols (15). De Man, Rogosa, and Sharpe (MRS, pH = 6.2–6.4) broth supplemented with 0.05% (w/v) L-cysteine was used for LAB activation or propagation when appropriate. Basal MRS medium (i.e., MRS without carbon source, bMRS) was prepared in accordance with previous protocol (16), and used for the analysis of inulin metabolic characteristics of the selected strain. The liquid bMRS containing 30 mg/L bromocresol purple was used for inulin fermentation testing by purified lactic acid bacteria strains.

### Sample Collection

October 2020, a total number of 27 Rex rabbits including weaned rabbits (*n* = 14) and suckling rabbits (*n* = 13) were randomly selected from a farm belonging to the Sichuan Academy of grassland sciences (Chengdu, China). The feces were collected aseptically, placed in a 10 mL sterile centrifuge tube, and transported to the laboratory in an icebox.

### Isolation and Identification of *Lactobacillus* Species

Prior to the isolation of *Lactobacillus* species, we started by enrichment of fresh rabbit feces in MRS broth anaerobically overnight at 37°C. Following the enrichment, aliquots from bacterial culture were 10-fold serially diluted in physiological saline. Diluted bacterium suspensions (100 μL) were plated onto LAMVAB medium followed by incubation for 48 h at 37°C anaerobically for *Lactobacillus* species isolation. Presumptive colonies with different morphology were picked and further streaked onto MRS plates twice consecutively to obtain pure bacterial isolates. The bacterial identity was confirmed by amplifying the 16S rRNA gene using universal primers 27F 5′-AGAGTTTGATCCTGGCTCAG-3′ and 1492R 5′-GGTTACCTTGTTACGACTT-3′. The PCR reaction was carried out in a total volume of 50 μL including 25 μL 2× Taq PCR Master Mix,1 μL DNA template, 2 μL each primer, and 20 μL sterilized ddH_2_O. The amplification was performed using a T100 Thermal Cycler (Bio-Rad, Beijing, China). All DNA was amplified for 35 cycles with an initial denaturation at 94°C for 4 min and a final extension at 72°C for 10 min. The program cycle consisted of 30 s denaturation at 94°C, 30 s annealing at 55°C, and 1.5 min extension at 72°C. PCR amplified DNA fragments were separated by electrophoresis in a 1.5% agarose gel at 105 V for 30 min and visualized after Goldview staining using the ChemiDoc™ XRS + System (Biorad, California, USA). Product size was determined by comparison with a 100~5,000 bp DNA ladder (Sangon Biotech, Shanghai, China). The resulting PCR products were sequenced by Tsingke (Chengdu, China). The sequences were trimmed for quality and uploaded to the BLAST database (NCBI) to determine their identity (>97% similarity).

### Evaluation of Inulin-Fermenting Capability of Isolates

Liquid bMRS containing bromocresol purple sodium salt (30 mg/L) supplemented with 1% inulin (bMRS-inulin), 1% fructo-oligosaccharides (bMRS-FOS), 1% glucose (bMRS-glucose), or water, respectively, were used to examine *in vitro* fermentability of inulin by rabbit gut-derived LAB. Specifically, activated bacterial cell culture (1%, v/v) was inoculated into bMRS-inulin or bMRS-FOS, using bMRS-glucose as a positive control and bMRS-H_2_O (bMRS without sugar) as a negative control, respectively. All incubations were carried out at 37°C anaerobically for 48–72 h. A color change from purple to yellow in the media as indicated by bromocresol purple was considered as a positive strain capable of metabolizing and utilizing inulin or FOS. Furthermore, the final bacterial cultures were subjected to pH determination and TLC analysis.

### Growth Characteristics Analysis of Positive Strains

Bacterial growth curves determination was carried out through inoculation of bacterial culture into basal MRS containing inulin, glucose, or water, respectively. Basal MRS containing 1% (w/v) inulin was used for determining the growth of a positive strain with inulin as the only carbon source. Basal MRS medium added with the same volume of sterile water was employed as the negative control, while bMRS containing glucose at 1% (w/v) was employed as the positive control. The effect of inulin on the growth of a positive strain was investigated by inoculating the overnight culture (1%, v/v) into bMRS containing 1% (w/v) glucose, 1% (w/v) inulin, and sterile ddH_2_O, respectively, then cultivating at 37°C for 48 h. Three-milliliter bacterial cultures were taken at 0, 1, 2, 3, 4, 6, 8, 10, 12, 14, 15, 20, 24, 30, 36, and 48 h, respectively for the determination of inulin metabolic characteristics of strains, including the pH of bacterial cultures during fermentation with a Leici pH meter PHBJ-206 (Shanghai, China) and the cell growth *via* optical density at 600 nm (OD_600nm_) using a spectrophotometer (Thermo Scientific™ Biomate™ 3S Spectrophotometer).

### Thin-Layer Chromatography Analysis

The dynamic changes of inulin components during fermentation by positive strains were analyzed through thin layer chromatography (TLC) following published protocols with minor modifications (16). The supernatant for each sample was obtained by centrifugation of bacterial cultures at 12,000 rpm in an Eppendorf centrifuge (Hamburg, Germany) for 2 min, then spotted twice (8 μL) onto glass plates pre-coated with silica gel GF_254_. The 1% Inulin or 1% FOS (8 μL) and 1% glucose standards (4 μL) were used as controls. TLC glass plates were developed twice with a mixture of n-butanol, acetic acid, and water at a ratio of 2:2:1 (v/v). TLC bands were visualized by staining with a solvent consisting of 2 g diphenylamine, 2 mL aniline, and 10 mL phosphoric acid (wt% = 85%) in 100 mL acetone, followed by air drying at 105°C for 5 min.

### Genomic DNA Extraction and Sequencing

The positive selected strain was cultured in liquid MRS medium at 37°C overnight and bacterial cells were harvested by centrifugation of overnight cultures at 12,000 rpm for 5 min. Genomic DNA was extracted from harvested cells using a Wizard Genomic DNA Purification Kit (Promega, Madision, WI) in accordance with the manufacturer's instructions, and over 1 μg high-quality genomic DNA was sent to Shanghai Majorbio Bio-pharm Technology Co., Ltd. (Shanghai, China) for library preparation and sequencing. A paired-end library was constructed using NEXTflex® Rapid DNA-Seq kit (Bioo Scientific Cor. Austin, TX, USA). Paired-end sequencing (2 × 150 bp) was performed on Illumina Hiseq X (Illumina Inc., San Diego, CA, USA) using HiSeq X Reagent Kits according to the manufacturer's recommendations (www.illumina.com).

### Bioinformatics Analysis

Raw sequence data were filtered to obtain clean data using Fastp, these data were used for *de novo* assembly using the SOAPdenovo v2.04 to obtain genome sequences (contigs) (17). The genome sequences were subsequently analyzed on the Majorbio I-sanger Cloud Platform (www.i-sanger.com). Protein-coding genes (CDSs) were predicted using Glimmer v3.02 (18), and global protein function annotation was completed by blasting genes against the Clusters of Orthologous Groups (COGs) and Kyoto Encyclopedia of Genes and Genomes (KEGG) databases. Genome visualization was carried out using CGview software. Carbohydrate-active enzymes (CAZymes) annotations in obtained genome sequences were performed using CAZy database (http://www.cazy.org/). The genomic sequence of the positive strain was also queried for acquired antibiotic resistance genes using ResFinder 4.0 with default settings.

### Phylogenetic Analysis

The phylogenetic tree based on 16S rRNA gene sequences was constructed with MEGAX software to study the taxonomic clustering of bacterial isolates (19). In detail, multiple sequence alignments were performed with the ClustalW program, and a phylogenetic tree was constructed through analysis of the agligned sequence by the neighbor-joining method. To evaluate the reliability of the tree, phylogenies were tested with 1,000 bootstrap replicates.

### Strain Deposition and Data Availability

The sequence data for *L. paracasei* YT170 genome and 16S rRNA gene have been deposited at GenBank database under the accession numbers JAFELK000000000 and MW577649, respectively. The strain has been deposited at the China General Microbiological Culture Collection Center (CGMCC no. 22089).

## Results

### Isolation and Identification of Lactobacilli From Young Rabbits

Isolation and identification of lactobacilli derived from twenty-seven rabbit fecal samples were carried out using the selective LAMVAB medium. The sampling information is shown in Supplementary Table 1. Consequently, a total number of 94 LAB strains were obtained from 19 young rabbit fecal samples, including 56 strains isolated from weaned rabbit fecal samples (*n* = 11) and 38 strains isolated from suckling rabbit fecal samples (*n* = 8). No LAB was recovered from eight fecal samples, including three fecal samples from weaned rabbits (TF08, TF10, and TF27) and five fecal samples from suckling rabbits (TF02, TF04, TF06, TF07, and TF26). On the basis of the 16S rRNA gene sequencing and analysis, the isolates were identified as *Lactiplantibacillus plantarum* (*n* = 32), *Pediococcus acidilactici* (*n* = 14), *Weissella kandleri* (*n* = 14), *Levilactobacillus brevis* (*n* = 7), *Lactobaillus ginsenosidimutans* (*n* = 7), *Lacticaseibacillus paracasei* (*n* = 7), *Latilactobacillus curvatus* (*n* = 6), *Weissella paramesenteroides* (*n* = 5), and *Weissella cibaria* (*n* = 2), respectively (Supplementary Table 2).

### Inulin Consumption by Rabbit-Derived LAB Isolates

All 94 LAB isolates were initially evaluated for their capability to ferment inulin and the results showed that only 28 strains (29.8%, 28/94) could metabolize and utilize inulin, including *L. plantarum* (*n* = 14), *L. paracasei* (*n* = 7), *W. paramesenteroides* (*n* = 3), *L. brevis* (*n* = 3), and *L. curvatus* (*n* = 1) (Table 1 and Supplementary Table 2). These positive strains were further subjected to fermentation tests using inulin only containing long-chain components validated by TLC analysis, and the results showed that only seven *L. paracasei* strains—all recovered from the same fecal sample, TF23—could grow well on the medium containing long-chain inulin as the carbon source (Supplementary Table 3). After 72 h of fermentation in bMRS medium containing 1% long-chain inulin (lc-inulin), the OD_600nm_ and pH values of all these seven strains could reach about 1.9 and declined to about 4.2, respectively, similar to those in bMRS medium containing 1% glucose (Supplementary Table 4). As a consequence, we selected *L. paracasei* YT170 as a representative of these positive strains for further research.

**Table 1:** Lactic acid bacteria recovered from young rabbits and their inulin fermentation phenotype.

**Species**	**All isolates**	**Positive isolates**
*Lactiplantibacillus plantarum*	32	14
*Pediococcus acidilactici*	14	0
*Weissella kandleri*	14	0
*Levilactobacillus brevis*	7	3
*Lactobaillus ginsenosidimutans*	7	0
*Lacticaseibacillus paracasei*	7	7
*Latilactobacillus curvatus*	6	1
*Weissella paramesenteroides*	5	3
*Weissella cibaria*	2	0
Total	94	28

By contrast, the other twenty-one positive strains prefered to utilize short-chain FOS components present in the inulin. In order to validate this speculation, fermentation tests were carried out with bMRS-FOS medium containing FOS as the carbon source for these strains. The results are shown in Supplementary Table 4 and we can see that all these strains could grow well on bMRS-FOS medium; final OD_600nm_ values of all these 28 strains after 72 h of fermentation were within the range of around 0.5 to 1.6; the values of pH declined to the range of 4.48–6.63. TLC analysis of bacterial culture supernatants after 72 h of fermentation by these 28 strains showed that strains including *L. plantarum* YT142, YT145, YT146, YT148, YT149, YT150, YT158, YT159, and YT160 were almost capable of degrading all components present in the FOS, while there were a few components with a low degree of polymerization (DP) remaining in the bacterial culture supernatants for all *L. paracasei* strains and a considerable amount of components with a wide range of DP for strains such as *W. paramesenteroides* YT174, YT175, and YT176 (Supplementary Figure 1).

### Inulin Metabolic Characteristics of *L. paracasei* YT170

**Figure 1:**
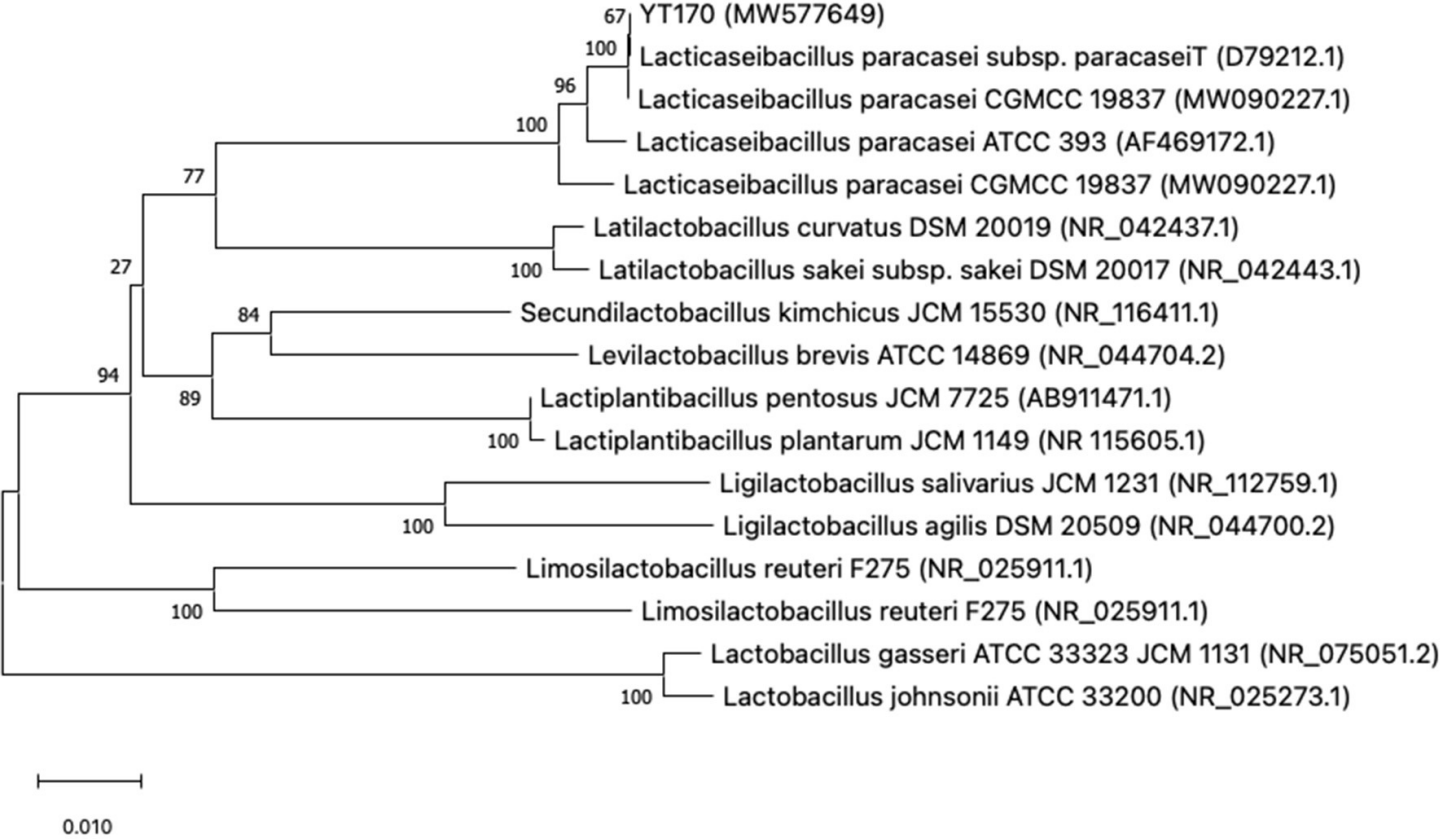
Phylogenetic tree of strain YT170 and related species constructed on the basis of 16S rRNA gene sequences using the neighbor-joining method. The numbers next to the branches indicate percentage values for 1,000 bootstrap replicates. GenBank accession numbers for the sequences are shown in parentheses. The scale bar represents 0.010 substitutions per site.

The colony and cell morphology of YT170 are shown in Supplementary Figure 2. The strain YT170 can form milky white, rounded, smooth, and nontransparent colonies with a size of about 1 mm in diameter, and the cells are gram-positive and typically rod-shaped. A phylogenetic tree was constructed using MEGAX software to determine the evolutionary relationship of strain YT170 (Figure 1). Strain YT170 showed the highest 16S rRNA gene sequence similarity with *Lacticaseibacillus paracasei* CGMCC 19837 (GenBank no. MW090227.1) and *Lacticaseibacillus paracasei* subsp. *paracasei*^T^ (GenBank no. D79212.1). Therefore, strain YT170 was identified as *Lacticaseibacillus paracasei* species.

**Figure 2:**
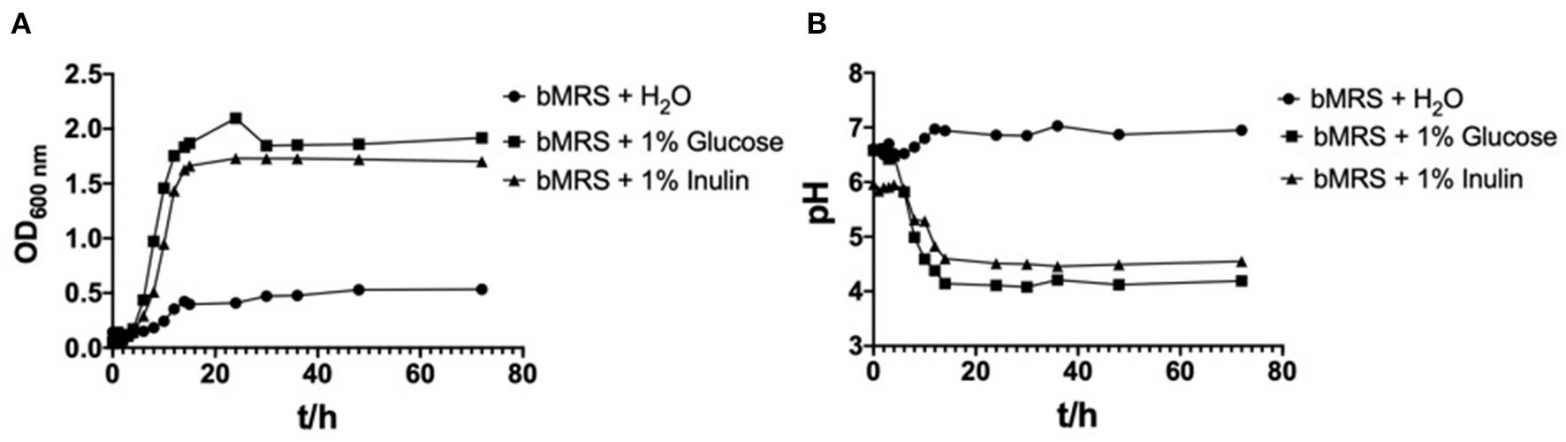
Growth curves of strain *Lacticaseibacillus paracasei* YT170 in bMRS medium containing 1% inulin and 1% glucose **(A)** and the pH change **(B)**.

The OD_600nm_ and pH values for *L. paracasei* YT170 with the incubation time in bMRS supplemented with the different carbon sources (inulin and glucose) and water respectively are presented in Figure 2. *L. paracasei* YT170 strain could grow on 1% inulin with final OD_600nm_ of 1.917 after 72 h close to the growth on 1% glucose with final OD_600nm_ of 1.701 (Figure 2A). The exponential phases of *L. paracasei* YT170 in bMRS with 1% glucose or inulin were both observed from the 4th to 14th hour. The fermentation of both inulin and glucose by *L. paracasei* YT170 significantly decreased the pH of bacterial cultures (Figure 2B). The pH of *L. paracasei* YT170 in bMRS with 1% inulin and glucose dropped from 6.58 to 4.55 and 4.19, respectively after 72 h of fermentation.

**Figure 3:**
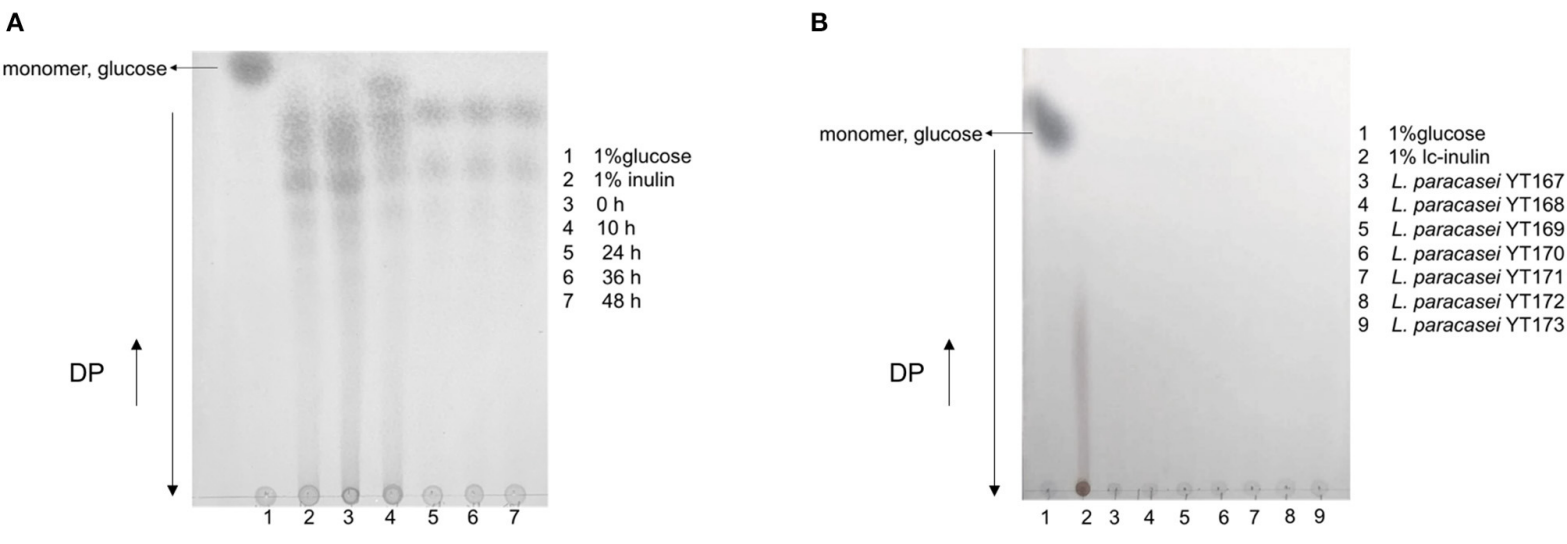
TLC profiling of bacterial culture supernatants at several sampling time-points during fermentation in bMRS broth containing 1% inulin by fermenter *Lacticaseibacillus paracasei* YT170 **(A)** and bacterial culture supernatants of long-chain inulin (lc-inulin) by seven *L. paracasei* strains after 72 h **(B)**. Monomer sugar, glucose, and degree of polymerization (DP) are marked with arrows.

The results of TLC analysis of bacterial culture supernatants of *L. paracasei* YT170 grown in medium with inulin as the carbon source are shown in Figure 3. In accordance with the growth characteristics of *L. paracasei* YT170, we selected samples taken at the sampling time-points of 0, 10, 24, 36, and 48 h for TLC analysis to understand the inulin consumption patterns. The results showed that oligofructose of moderate chain length disappeared after 10 h of fermentation by *L. paracasei* YT170, and short-chain oligofructose with a degree of polymerization (DP) ranging from around 2 to 6 was also consumed to some extent. After 24 h of growth, all moderate and long-chain inulin components were completely consumed by *L. paracasei* YT170, and only a minimal short-chain oligofructose (DP 2–6) was left in the medium (Figure 3A). In addition, TLC analysis was carried out for *L. paracasei* YT170 together with the other six *L. paracasei* strains capable of metabolizing and utilizing long-chain inulin (lc-inulin). This was done using bacterial culture supernatants after 72 h of fermentation in bMRS containing only long-chain fructan components, and the results showed that all lc-inulin components were completely utilized after 72 h of fermentation by all these seven strains (Figure 3B).

### Genetic Analyses of Inulin Consumption by *L. paracasei* YT170

**Figure 4:**
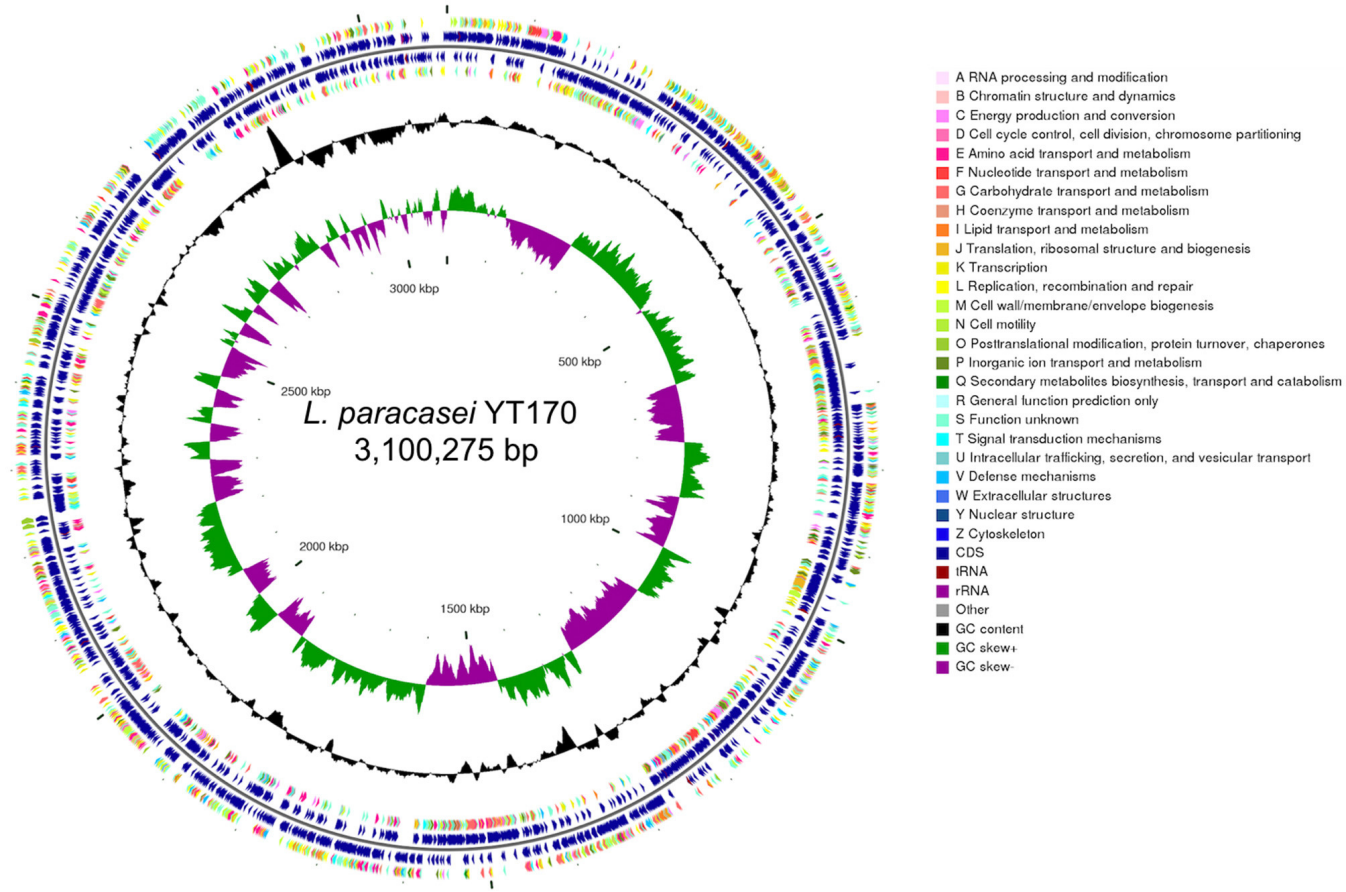
Genome features of *L. paracasei* YT170. circular genomic map of *L. paracasei* YT170. The circular map was generated using CGview and contains seven circles. From the inner circle to the outer ring: The first lap depicts the location of the genome. The second lap shows the GC skew (G+C/G–C), which the value is plotted as the deviation from the average GC skew of the complete sequence. The third lap shows GC content. The 5th (forward strand) and 6th lap (reverse strand) indicate the sites of CDSs/rRNA/tRNA on the genome. The 4th (forward strand) and 7th lap (reverse strand) illustrate the CDSs, colored according to COG function categories.

Whole-genome analysis was carried out through genomic DNA extraction, sequencing on Illumina Hiseq X, and bioinformatics analysis. As shown in Figure 4, whole-genome sequencing and genome assembly resulted in 138 scaffolds with an N50 value of 56,807 bp and a sequencing depth of 330 ×; the genome of *L. paracasei* YT170 is further assembled to a size of 3.10 M, with GC content of 46.22%. A total of 3,077 protein-coding genes (CDSs) were predicted in the genome of *L. paracasei* YT170, accounting for 83.04% of the whole genome.

**Figure 5:**
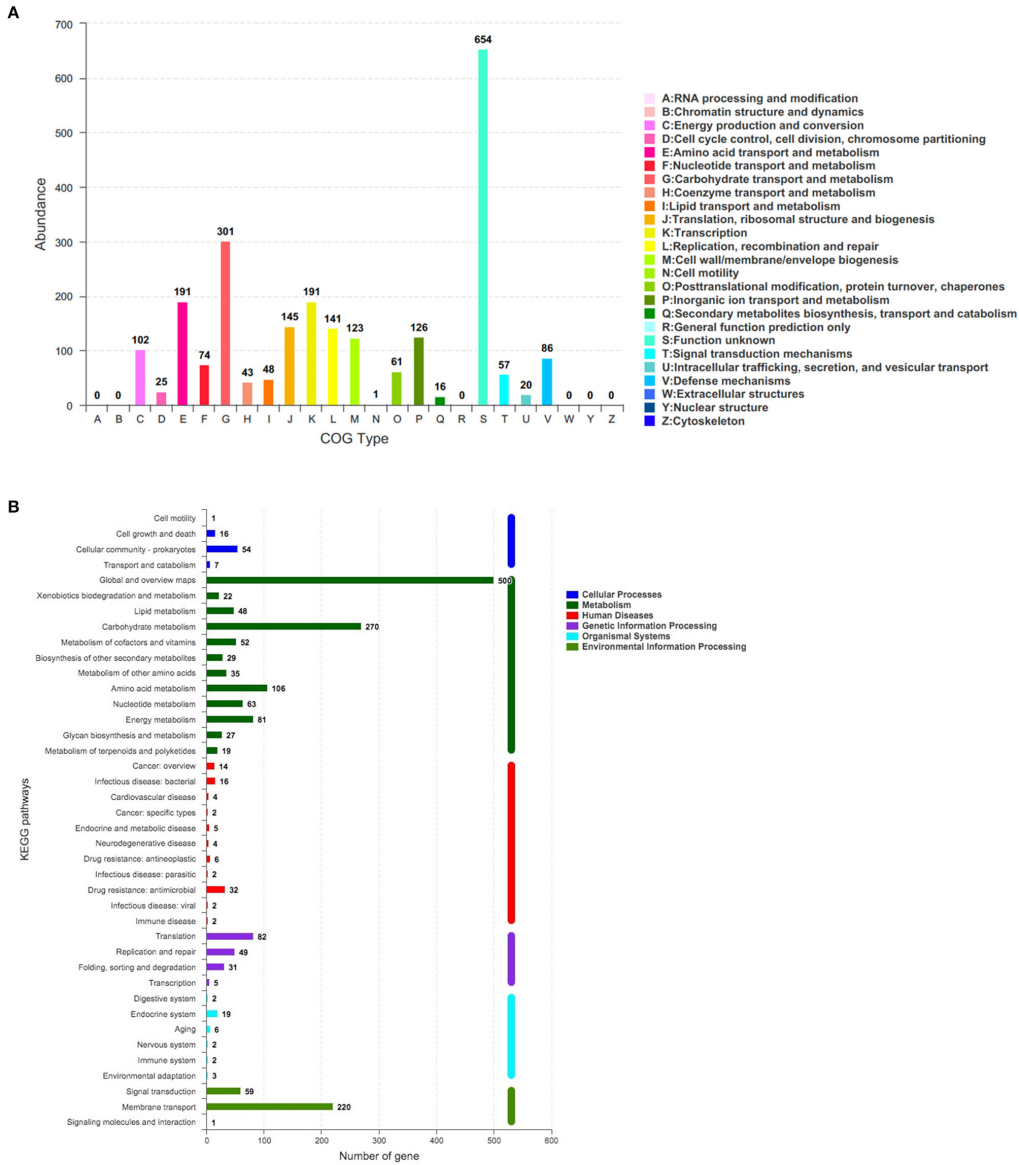
The number of genes assigned to COGs **(A)** and KEGG **(B)** function categories in the genome of *L. paracasei* YT170. COGs, Clusters of Orthologous Groups (COGs) database; KEGG, Kyoto Encyclopedia of Genes and Genomes (KEGG) database.

The corresponding function annotations of these CDSs were completed by blasting genes against both COG and KEGG databases. Consequently, a total of 2,376 CDSs (2,376/3,077, 77.22%) were specifically assigned to clusters of COG families comprising nineteen function categories (Figure 5A), among which 301 genes (12.67%, 301/2,376) were classified into function categories for carbohydrate transport and metabolism. In addition, a total of 1,501 CDSs (1,501/3,077, 48.78%) were classified into six KEGG function categories and 36 functional pathways (Figure 5B), mainly functioning in the global and overview maps (500 genes), carbohydrate metabolism (270 genes), membrane transport (220 genes), and amino acid metabolism (106 genes).

Furthermore, the genome-wide analysis of CAZymes revealed that *L. paracasei* YT170 genome contained 94 genes encoding CAZymes including carbohydrate esterase (CE), glycosyl transferase (GT), glycoside hydrolase (GH), and auxiliary activity (AA) (Supplementary Table 5). In the genome of *L. paracasei* YT170, gene1319 encodes a putative β-fructofuranosidase (EC 3.2.1.26) containing 492 amino acids belonging to the GH32 family, and gene1410 encodes a putative fructan β-fructosidase (EC 3.2.1.80) consisting of 1,375 amino acids and containing a conseved domain belonging to the GH32 family. The deduced amino acid sequences of the fructan β-fructosidase encoded by gene1319 reveal that it comprises of a long and complicated region for cell-wall anchoring, consisting of two peptidoglycan binding domains Big3 in addition to the GH32-family catalytic part (Supplementary Figure 3).

**Figure 6:**
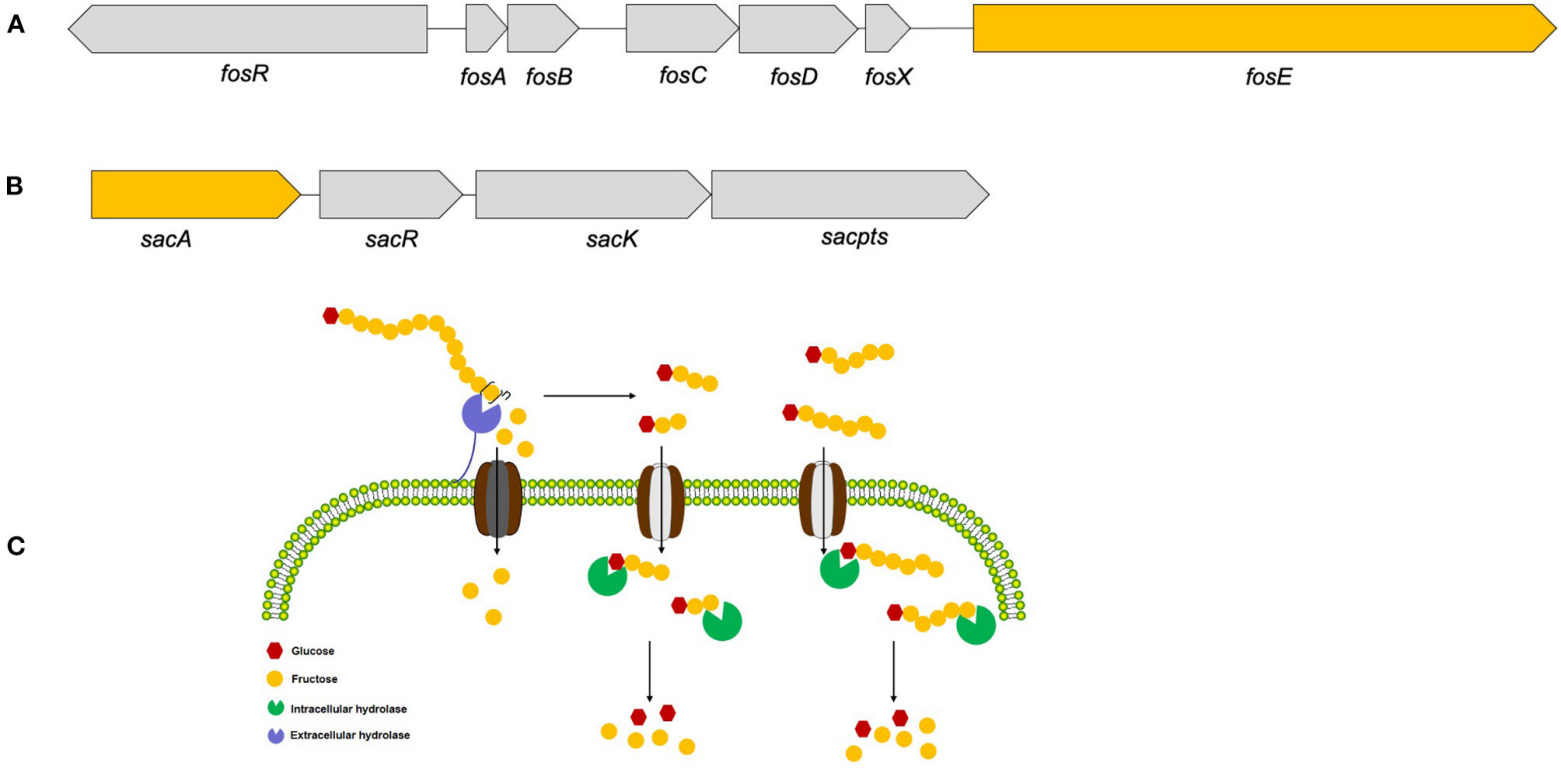
The two gene clusters predicted to link to inulin consumption **(A,B)** and the proposed metabolic routes of inulin **(C)** by *L. paracasei* YT170.

Therefore, based on these functional annotations of strain YT170 genome, a 6.12 kb gene cluster comprising four genes (gene1319, gene1320, gene1321, and gene1322: gene cluster no. 1) (Figure 6A) and another 9.576 kb gene cluster consisting of seven genes (gene1404, gene1405, gene1406, gene1407, gene1408, gene1409, and gene1410: gene cluster no. 2) were predicted to link to inulin fermentation (Figure 6B). Gene cluster no. 1 consists of *fosR* (gene1404) encoding a transcriptional regulator (fosR), five genes including *fosA* (gene1405), *fosB* (gene1406), *fosC* (gene1407), *fosD* (gene1408), and *fosX* (gene1409) encoding EIIA, IIB, IIC, IID, and EII component of a fructose/mannose-specific PTS, and *fosE* (gene1410) encoding a fructan β-fructosidase. This gene cluster was similar to the reported *fosRABCDXE* operon in strains *L. paracasei* 1195 and *L. casei* ATCC 334 in terms of both the structural organization and sequence similarity (20). The product of gene1404 exhibited 99.88 and 99.05% amino acid sequence identity with the transcriptional regulator fosR of *L. paracasei* 1195 (Genebank accession no. ABD57313.1) and a putative transcriptional regulator present in levanase operon of *L. casei* ATCC 334 (Genebank accession no. ABJ69406.1), respectively; the fructan β-fructosidase encoded by gene1410 present in the genome of *L. paracasei* YT170 showed sequence similarity of 97.85 and 74.94% with those of *L. paracasei* 1195 (Genebank accession no. ABD57319.1) and *L. casei* ATCC 334 (Genebank accession no. ABJ69412.1) respectively (Supplementary Figure 4); the amino acid sequences of five components of fructose/mannose-specific PTS encoded by gene1405, gene1406, gene1407, gene1408, and gene 1409 showed similarity of more than 99% with those of *L. paracasei* 1195 and *L. casei* ATCC 334.

Moreover, gene cluster no. 2 found in the draft genome sequences of strain YT170 consists of *sacA* (gene1319), *sacR* (gene1320), *sacK* (gene1321), and *sacpts* (gene1322). In detail, gene1322 was predicted to encode a PTS sugar transporter subunit EIIBCA, and gene1319 encoded a putative β-fructofuranosidase which showed amino acid sequence similarity of 44.38% with sucrose-6-phosphate hydrolase (Genebank accession no. CCC77729.1) in FOS-fermenter *L. plantarum* WCFS1 (21) and 44.17% with beta-fructofuranosidase (Genebank accession no. YP_003923471.1) in *L. plantarum* subsp. *plantarum* ST-III (22) (Supplementary Figure 5). This gene cluster was similar to the structure of the *pts1BCA* operon in which there was a sucrose PTS transport system involved in transporting scFOS in *L. plantarum* WCSF1 in conjunction with an intracellular beta-fructofuranosidase encoded by *sacA*.

## Discussion

This study is the first attempt to systematically investigate lactobacilli from neonate rabbits through isolation and identification of pure strains from fecal samples collected from suckling and weaned rabbits. *Lactobacillus* (*n* = 59), *Weissella* (*n* = 21), and *Pediococcu*s (*n* = 14) species were three major lactic acid bacteria recovered from both weaned and suckling rabbits through the selective medium LAMVAB which was previously reported for selective lactobacilli isolation due to its low pH and the presence of vancomycin (15). In accordance with previous research, this medium is not useful as a selective agent in samples containing vancomycin-resistant species *Pediococci, Weissella*, or *Pediococcus* species, etc. (15) which is consistent with the results of the present study that *Weissella* and *Pediococcu*s species were also recovered from young rabbit fecal samples in addition to *Lactobacillus* species. In addition, all nine purified species belonging to the *Weissella, Pediococcu*s, and *Lactobacillus* genera were recovered from weaned rabbits; in contrast, only five species were recovered from suckling rabbits, which indicated there might be higher diversity of lactic acid bacteria in the gut of weaned rabbits in comparison with suckling rabbits on this farm. To date, the research about the isolation and identification of beneficial microbes such as LAB from rabbits was little reported. In addition, few LAB strains derived from rabbit gut were used for the LAB administration in rabbits in previous publications (23–25). In terms of co-evolution between the host immune system and the gastrointestinal microbiome, promising probiotics should derive from the gut of host animals, which could take the advantage of being able to adapt to the harsh gastroenteric environments and further achieve stable colonization in the gut and exert their beneficial effects (26). Therefore, the present study for the first time obtained a variety of LAB strains from young rabbits, which could be extremely important to provide microbial resources for candidate probiotics in the development and application of antibiotic alternatives specifically for rabbits in the future.

After obtaining pure LAB isolates, we carried out the fermentation tests by using the medium containing inulin as the carbon source. In accordance with our results, lactic acid bacteria capable of metabolizing inulin represented a low proportion of the total population while non-responders were present in the majority of fecal samples collected from young rabbits in this specific farm. Therefore, we can speculate that only some species or strains can efficiently respond to inulin, and inulin conversely promotes the growth and proliferation of these lactic acid bacteria and further contributes to their survival and colonization in the intestine of rabbits should we carry out oral inulin administration in neonate rabbits. To date, this study first evaluated the capability of LAB isolated from the intestinal flora of neonate rabbits to metabolize the common commercial prebiotic, inulin, thus making clear responders and non-responders in the gut which is quite critical for the application of prebiotic, specifically inulin, in modulating gut microbiota of neonate rabbits in the early life.

To date, several studies have shown that a few *L. plantarum* and *L. paracasei* strains could ferment and utilize inulin as the sole carbon source (27, 28), but there are few reports on the metabolization and utilization of inulin by *W. paramesenteroides, L. brevis*, and *L. curvatus* which were found to be capable of growing very well in medium containing inulin as the carbon source in the present study. Only two *W. paramesenteroides* strains isolated from fruits were found to be capable of utilizing the low molecular weight of fructooligosaccharides (FOS) (29). Also, the capability of inulin metabolism and utilization by lactic acid bacteria from the gut of young rabbits showed species difference; for instance, all strains belonging to *P. acidilactici, W. kandleri, L. ginsenosidimutans* and *W. cibaria* could not grow in a bMRS medium containing inulin as the sole carbon source, while several strains belonging to *L. plantarum, L. paracasei*, and *L. brevis* grew very well-through inulin fermentation. Furthermore, the inulin fermentation phenotype of these lactic acid bacteria strains from young rabbits was also strain-specific; for instance, in bMRS medium containing inulin with scFOS, only 43.75% of *L. plantarum* isolates (14/32), 42.86% (3/7) of *L. brevis* strains, 16.67% (1/6) of *L. curvatus* strains, and 60% (3/5) of *W. paramesenteroides* strains were capable of fermenting inulin well. In addition, among 28 lactic acid bacteria strains capable of growing well on bMRS medium with 1% inulin containing scFOS, only seven of them could ferment and utilize long-chain inulin in which no scFOS was available to these strains, which indicated that these lactic acid bacteria from young rabbits showed a structure-activity relationship in metabolizing and utilizing inulin. These seven strains capable of degrading long-chain inulin were all *L. paracasei* species and purified from the same fecal sample; therefore, we selected *L. paracasei* YT170 as a representative of most vigorous inulin-degrading strains for further research.

After obtaining the most vigorous inulin-degrading strain *L. paracasei* YT170, we investigated the inulin metabolic characteristics of the strain YT170; as a consequence, on the basis of the growth curves and pH change, *L. paracasei* YT170 was capable of efficiently consuming inulin, showing potential to be used as a tailored synergistic synbiotic with a combination of inulin aimed at modulating intestinal microbiota of neonate rabbits in early life. In addition, TLC analysis indicated that *L. paracasei* YT170 was capable of metabolizing and utilizing both short-chain oligofructose and long-chain fructan components, which suggested that there might be functional genes encoding extracellular inulin-degrading enzymes in the genome of *L. paracasei* YT170.

In order to figure out inulin consumption related functional genes in *L. paracasei* YT170, whole-genome sequencing and bioinformatics analysis were carried out and both COG and KEGG functional annotations indicated that rabbit gut-derived *L. paracasei* YT170 harbored a high percentage of functional genes related to carbohydrate transporter and metabolism, which was consistent with the previous research in which it has been proposed that gut-associated strains have adapted to their niche with a specialized set of carbohydrate-metabolizing genes, encoding proteins including hydrolases and transporters (30). Particularly, functional annotation against the CAZy database revealed that there were 39 glycoside hydrolases responsible for breaking down carbohydrates into smaller products indicating that *L. paracasei* YT170 possesses the capability to degrade carbohydrates, which might contribute to the survival of the strain YT170 in the gut of neonate rabbit through carbohydrate metabolism. Among these 39 glycoside hydrolases (GH) encoding genes, two genes encode putative fructan β-fructosidase and β-fructofuranosidase, respectively, which both belong to the glycoside hydrolase family 32 (GH32). As for the former, the deduced amino acid sequences of the fructan β-fructosidase encoded by gene1410 revealed that this putative inulin-degrading enzyme comprises of a long and complicated region for cell-wall anchoring, consisting of two Big3 domains for peptidoglycan binding, in addition to a GH32-family catalytic part (31), indicating the putative fructan β-fructosidase is a cell wall anchored extracellular inulin-degrading enzyme, which was consistent with our above speculation that long-chain inulin components might be extracellularly degraded on the basis of the TLC analysis. In accordance with previous research, GH32 and GH68 families target fructan degradation, such as bacterial endo and exo-inulinases, levanases, and β-fructofuranosidases (32), indicating the potential of these two genes present in the genome of *L. paracasei* YT170 for degrading fructosyl units [beta-() linkage] in inulin (33). Therefore, on the basis of above functional annotation results of *L. paracasei* YT170 genome, in total, two metabolic routes, completed by the *fosRABCDXE* and *pts1BCA* operons, might be involved in inulin metabolism of *L. paracasei* YT170: in the first route *L. paracasei* YT170 degraded long-chain inulin components, fructan, outside the cell through the extracellular fructan β-fructosidase, releasing short-chain fructooliogsaccharides or monomer, fructose, which were transported by fructose/mannose-specific PTS into the cytoplasm and completely metabolized; in the second route, *L. paracasei* YT170 transported scFOS through the PTS sugar transporter into the cytoplasm and degraded it using intracellular beta-fructofuranosidase (Figure 6C) (34). Finally, we analyzed the YT170 genome sequence against the Resfinder database and no transferable resistance genes were predicted in the genome, indicating the strain YT170 could be applied to neonate rabbits as a safe probiotic in terms of preventing transference of antibiotic resistance genes.

## Conclusion

In conclusion, this study firstly recovered 94 lactic acid bacteria strains belonging to various species from young rabbits and further found that only some species or strains can efficiently respond to inulin, and inulin conversely promotes the growth and proliferation of these lactic acid bacteria strains from young rabbits in this specific farm, indicating both significant species- and strain- specificity. One inulin-degrading strain, *L. paracasei* YT170, could efficiently utilize both short-chain and long-chain inulin components, and a high percentage of genes present in the genome of rabbit gut-derived *L. paracasei* YT170 were assigned to the function categories of carbohydrate transporter and metabolism. Assignment of CAZy family to a sequence using similarity search against the CAZy database revealed that 39 glycoside hydrolase (GH) encoding genes are present in the genome and 2 genes encoding putative β-fructofuranosidase and extracellular fructan β-fructosidase, respectively belonging to GH32 family might relate to inulin degradation. As a consequence, one gene cluster *fosRABCDXE* operon related to extracellular degradation, and another gene cluster *pts1BCA* operon related to intracellular metabolism, were predicted to link to inulin consumption by *L. paracasei* YT170; however, the proposed metabolic routes and molecular mechanisms might require further exploration through transcriptomics and molecular biological methods such as gene knockouts, etc.

## Data Availability Statement

The datasets presented in this study can be found in online repositories. The names of the repository/repositories and accession number(s) can be found in the article/Supplementary Material.

## Ethics Statement

The animal study was reviewed and approved by Sichuan Normal University Ethic Committee.

## Author Contributions

Y-tZ and R-tL designed the experiments and drafted the manuscript. Y-tZ, YW, JY, and R-tL carried out most of the experiments and analyzed the data. K-jL carried out sample collection. S-mY, YL, S-xQ, and Z-YX revised the manuscript. YZ provided a guarantee for the smooth running of the entire trial. Y-tZ, S-mY, and R-tL had primary responsibility for the final content. All authors contributed to the article and approved the submitted version.

## Funding

We thank the funding supports including research expenditure provided by the Applied Fundamental Research Project of Science and Technology Department of Sichuan Province (Grant No. 2021YJ0274) and Scientific Research Foundation of Sichuan Normal University (Grant No. XJ20200175), and open access publication fees provided by Doctoral Scientific Research Foundation of Sichuan Water Conservancy College (Grant No. 2021SCSZYD-02).

## Conflict of Interest

The authors declare that the research was conducted in the absence of any commercial or financial relationships that could be construed as a potential conflict of interest.

## Publisher's Note

All claims expressed in this article are solely those of the authors and do not necessarily represent those of their affiliated organizations, or those of the publisher, the editors and the reviewers. Any product that may be evaluated in this article, or claim that may be made by its manufacturer, is not guaranteed or endorsed by the publisher.

## References

[1] Cullere M Dalle Zotte A. Rabbit meat production and consumption: state of knowledge and future perspectives. Meat Sci. (2018) 143:137–46. 10.1016/j.meatsci.2018.04.029 29751220

[2] Chen H-J Yang W-Y Wang C-Y. The review on structure of intestinal flora at different growth stages of rabbits. In: *International Conference on Medicine Sciences and Bioengineering (ICMSB 2017)*. (2017). p. 245–53. 10.12783/dtbh/icmsb2017/17972

[3] Kirchhelle C Pharming animals: a global history of antibiotics in food production (1935-2017). Palgrave Commun. (2018) 4:1–13. 10.1057/s41599-018-0152-2

[4] Beaumont M Paës C Mussard E Knudsen C Cauquil L Aymard P. Gut microbiota derived metabolites contribute to intestinal barrier maturation at the suckling-to-weaning transition. Gut Microbes. (2020) 11:1268–86. 10.1080/19490976.2020.1747335 32352849 PMC7524271

[5] Fang S Chen X Ye X Zhou L Xue S Gan Q. Effects of gut microbiome and short-chain fatty acids (SCFAs) on finishing weight of meat rabbits. Front Microbiol. (2020) 11:1835. 10.3389/fmicb.2020.01835 32849435 PMC7431612

[6] Combes S Fortun-Lamothe L Cauquil L Gidenne T. Engineering the rabbit digestive ecosystem to improve digestive health and efficacy. Animal. (2013) 7:1429–39. 10.1017/S1751731113001079 23769161

[7] Falcão-e-Cunha L Castro-Solla L Maertens L Marounek M Pinheiro V Freire J. Alternatives to antibiotic growth promoters in rabbit feeding: a review. World Rabbit Sci. (2007) 15:127–40. 10.4995/wrs.2007.597

[8] Hernot DC Boileau TW Bauer LL Middelbos IS Murphy MR Swanson KS. *In vitro* fermentation profiles, gas production rates, and microbiota modulation as affected by certain fructans, galactooligosaccharides, and polydextrose. J Agric Food Chem. (2009) 57:1354–61. 10.1021/jf802484j 19199596

[9] Cheng W Lu J Li B Lin W Zhang Z Wei X. Effect of functional oligosaccharides and ordinary dietary fiber on intestinal microbiota diversity. Front Microbiol. (2017) 8:1750. 10.3389/fmicb.2017.01750 28979240 PMC5611707

[10] Chen Y Xie Y Zhong R Liu L Lin C Xiao L. Effects of xylo-oligosaccharides on growth and gut microbiota as potential replacements for antibiotic in weaning piglets. Front Microbiol. (2021) 12:355. 10.3389/fmicb.2021.641172 33717037 PMC7947891

[11] Ahmed W Rashid S. Functional and therapeutic potential of inulin: a comprehensive review. Crit Rev Food Sci Nutr. (2019) 59:1–13. 10.1080/10408398.2017.1355775 28799777

[12] Molina J Martorell J Hervera M Pérez-Accino J Fragua V Villaverde C. Preliminary study: fibre content in pet rabbit diets, crude fibre versus total dietary fibre. J Anim Physiol Anim Nutr. (2015) 99:23–8. 10.1111/jpn.12309 25865419

[13] Samanta A Jayapal N Senani S Kolte A Sridhar M. Prebiotic inulin: useful dietary adjuncts to manipulate the livestock gut microflora. Braz J Microbiol. (2013) 44:1–14. 10.1590/S1517-83822013005000023 24159277 PMC3804171

[14] Dokoupilova A Zita L Kvaček J Janda K Hofmanova B Masopustova R. Jerusalem artichoke (*Helinathus tuberosus*) tops as a natural source of inulin in rabbit diet: effect on growth performance and health status. J Cent Eur Agric. (2019) 20:796–801. 10.5513/JCEA01/20.3.2251

[15] Hartemink R Domenech V Rombouts F. LAMVAB-a new selective medium for the isolation of lactobacilli from faeces. J Microbiol Methods. (1997) 29:77–84. 10.1016/S0167-7012(97)00025-0

[16] Zhu Y Liu J Lopez JM Mills DA. Inulin fermentation by lactobacilli and bifidobacteria from dairy calves. Appl Environ Microbiol. (2020) 87:e01738–20. 10.1128/AEM.01738-20 33008824 PMC7755250

[17] Luo R Liu B Xie Y Li Z Huang W Yuan J. SOAPdenovo2: an empirically improved memory-efficient short-read *de novo* assembler. Gigascience. (2012) 1:18. 10.1186/2047-217X-1-18 23587118 PMC3626529

[18] Delcher AL Harmon D Kasif S White O Salzberg SL. Improved microbial gene identification with GLIMMER. Nucleic Acids Res. (1999) 27:4636–41. 10.1093/nar/27.23.4636 10556321 PMC148753

[19] Stecher G Tamura K Kumar S. Molecular evolutionary genetics analysis (MEGA) for macOS. Mol Biol Evol. (2020) 37:1237–9. 10.1093/molbev/msz312 31904846 PMC7086165

[20] Goh Y.J Zhang C Benson AK Schlegel V Lee J-H Hutkins RW. Identification of a putative operon involved in fructooligosaccharide utilization by *Lactobacillus paracasei*. Appl Environ Microbiol. (2006) 72:7518–30. 10.1128/AEM.00877-06 17028235 PMC1694223

[21] Saulnier DM Molenaar D De Vos WM Gibson GR Kolida S. Identification of prebiotic fructooligosaccharide metabolism in *Lactobacillus plantarum* WCFS1 through microarrays. Appl Environ Microbiol. (2007) 73:1753–65. 10.1128/AEM.01151-06 17261521 PMC1828832

[22] Chen C Zhou F Ren J Ai L Dong Y Wu Z. Cloning, expression and functional validation of a β-fructofuranosidase from *Lactobacillus plantarum*. Process Biochem. (2014) 49:758–67. 10.1016/j.procbio.2014.02.013

[23] Simonová MP Lauková A Žitnan R Chrastinová L. Effect of rabbit-origin enterocin-producing probiotic strain *Enterococcus faecium* CCM7420 application on growth performance and gut morphometry in rabbits. Czech J. Anim Sci. (2015) 60:509–12. 10.17221/8559-CJAS

[24] Wang C Zhu Y Li F Huang L. The effect of Lactobacillus isolates on growth performance, immune response, intestinal bacterial community composition of growing Rex Rabbits. J Anim Physiol Anim Nutr. (2017) 101:e1–e13. 10.1111/jpn.12629 28066944

[25] Zhou Y Ni X Wen B Duan L Sun H Yang M. Appropriate dose of *Lactobacillus buchneri* supplement improves intestinal microbiota and prevents diarrhoea in weaning Rex rabbits. Benef Microbes. (2018) 9:401–16. 10.3920/BM2017.0055 29380642

[26] Xiao Y Zhai Q Zhang H Chen W Hill C. Gut colonization mechanisms of *Lactobacillus* and *Bifidobacterium*: an argument for personalized designs. Annu Rev Food Sci Technol. (2021) 12:213–33. 10.1146/annurev-food-061120-014739 33317320

[27] Goh YJ Lee J-H Hutkins RW. Functional analysis of the fructooligosaccharide utilization operon in *Lactobacillus paracasei* 1195. Appl Environ Microbiol. (2007) 73:5716–24. 10.1128/AEM.00805-07 17644636 PMC2074902

[28] Buntin N Hongpattarakere T Ritari J Douillard FP Paulin L Boeren S. An inducible operon is involved in inulin utilization in *Lactobacillus plantarum* strains, as revealed by comparative proteogenomics and metabolic profiling. Appl Environ Microbiol. (2017) 83:e02402–16. 10.1128/AEM.02402-16 27815279 PMC5203619

[29] Pabari K Pithva S Kothari C Purama RK Kondepudi KK Vyas BRM. Evaluation of probiotic properties and prebiotic utilization potential of Weissella paramesenteroides isolated from fruits. Probiotics Antimicrob Proteins. (2020) 12:1126–38. 10.1007/s12602-019-09630-w 31942681

[30] O'sullivan O O'callaghan J Sangrador-Vegas A Mcauliffe O Slattery L Kaleta P. Comparative genomics of lactic acid bacteria reveals a niche-specific gene set. BMC Microbiol. (2009) 9:50. 10.1186/1471-2180-9-50 19265535 PMC2660350

[31] Velikova P Petrov K Petrova P. The cell wall anchored β-fructosidases of *Lactobacillus paracasei*: overproduction, purification, and gene expression control. Process Biochem. (2017) 52:53–62. 10.1016/j.procbio.2016.10.010

[32] Lammens W Le Roy K Schroeven L Van Laere A Rabijns A Van Den Ende W. Structural insights into glycoside hydrolase family 32 and 68 enzymes: functional implications. J Exp Bot. (2009) 60:727–40. 10.1093/jxb/ern333 19129163

[33] Goh YJ Klaenhammer TR. Genetic mechanisms of prebiotic oligosaccharide metabolism in probiotic microbes. Annu Rev Food Sci Technol. (2015) 6:137–56. 10.1146/annurev-food-022814-015706 25532597

[34] Petrova P Petrov K. Prebiotic-probiotic relationship: the genetic fundamentals of polysaccharides conversion by *Bifidobacterium* and *Lactobacillus* genera. In: Grumezescu AM Holban AM editor. Food Bioconversion., Pittsburgh: Academic Press (2017). p. 237–78.

