# Supplementary material for: Prebiotics Inulin Metabolism by Lactic Acid Bacteria From Young Rabbits
Source: Front Vet Sci. 2021 Oct 1;8:719927. doi: 10.3389/fvets.2021.719927 (PMC8517115; doi:10.3389/fvets.2021.719927)
Supplement: Supplementary file 1 [file Data_Sheet_1.PDF]

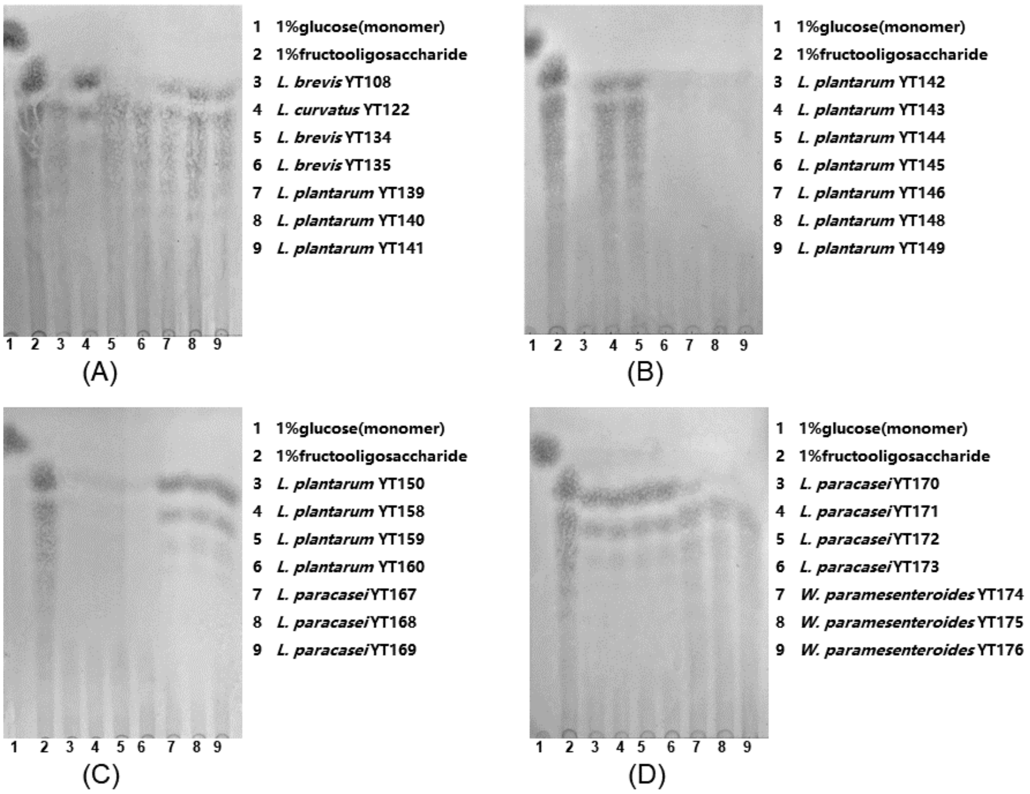

Supplementary Fig. S1. TLC results representing supernatants of fermented media after 72 h in bMRS broth containing 1% FOS by twenty-eight fermenters including seven strains (*L. brevis* YT108, *L. curvatus* YT122, *L. brevis* YT134, *L. brevis* YT135, *L. plantarum* YT139, *L. plantarum* YT140 and *L. plantarum* YT141; A), seven strains (*L. plantarum* YT142, YT143, YT144, YT145, YT146, YT148 and YT149; B), seven strains (*L. plantarum* YT150, YT158, YT159, YT160, and *L. paracasei* YT167, YT168, YT169; C), and seven strains (*L. paracasei* YT170, YT171, YT172, YT173, and *W. paramesenteroides* YT174, YT175, YT175; D).

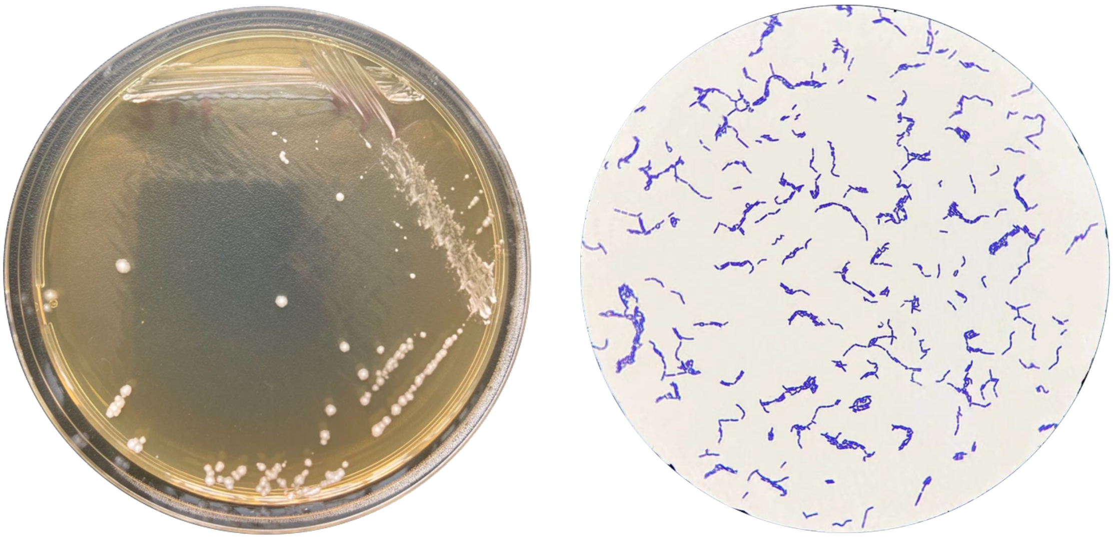

(A) (B)

Supplementary Fig. S2. The colony (A) and cell morphology (B, 10×100) of *L. paracasei* YT170

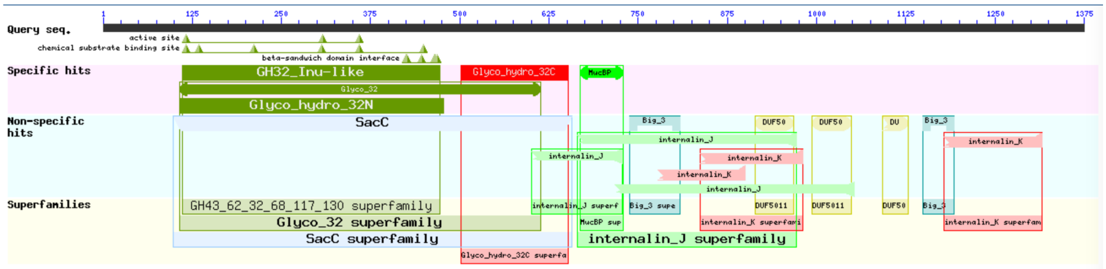

Name	Accession	Description	Interval	E-value
[+] GH32_Inu-like	cd18622	glycoside hydrolase family 32 protein such as Aspergillus ficuum endo-inulinase (Inu2); This ...	112-472	2.34e-118
[+] Glyco_32	smart00640	Glycosyl hydrolases family 32;	107-613	1.83e-92
[+] Glyco_hydro_32N	pfam00251	Glycosyl hydrolases family 32 N-terminal domain; This domain corresponds to the N-terminal ...	107-477	2.99e-83
[+] SacC	COG1621	Sucrose-6-phosphate hydrolase SacC, GH32 family [Carbohydrate transport and metabolism];	98-657	9.51e-83
[+] Glyco_hydro_32C	pfam08244	Glycosyl hydrolases family 32 C terminal; This domain corresponds to the C terminal domain of ...	502-652	7.57e-18
[+] MucBP	pfam06458	MucBP domain; The MucBP (MUCin-Binding Protein) domain is found in a wide variety of bacterial ...	668-729	1.19e-11
[+] Big_3	pfam07523	Bacterial Ig-like domain (group 3); This family consists of bacterial domains with an Ig-like ...	738-808	3.04e-08
[+] internalin_J	NF033187	class 1 internalin InlJ; Internalins, as found in the intracellular human pathogen Listeria ...	665-972	7.72e-08
[+] DUF5011	pfam16403	Domain of unknown function (DUF5011); This small family of proteins is functionally ...	914-968	3.48e-07
[+] DUF5011	pfam16403	Domain of unknown function (DUF5011); This small family of proteins is functionally ...	1092-1128	4.72e-06
[+] DUF5011	pfam16403	Domain of unknown function (DUF5011); This small family of proteins is functionally ...	994-1048	4.91e-06
[+] Big_3	pfam07523	Bacterial Ig-like domain (group 3); This family consists of bacterial domains with an Ig-like ...	1149-1192	1.21e-05
[+] internalin_K	NF033186	class 1 internalin InlK; Internalins, as found in the intracellular human pathogen Listeria ...	837-980	1.00e-04
[+] internalin_K	NF033186	class 1 internalin InlK; Internalins, as found in the intracellular human pathogen Listeria ...	1178-1316	4.41e-04
[+] internalin_K	NF033186	class 1 internalin InlK; Internalins, as found in the intracellular human pathogen Listeria ...	778-900	4.72e-04
[+] internalin_J	NF033187	class 1 internalin InlJ; Internalins, as found in the intracellular human pathogen Listeria ...	718-1052	2.72e-03
[+] internalin_J	NF033187	class 1 internalin InlJ; Internalins, as found in the intracellular human pathogen Listeria ...	600-728	3.85e-03

Supplementary Fig. S3. Scheme of functional structure of fructan β-fructosidase in *L. paracasei* YT170

```
CLUSTAL X (1.83) multiple sequence alignment


YT170           ------------------------------------------------------------
ABD57319.1      MEMDEKKHYKMYKSKSVWVFACLSTCLIVSFFNDGQNVSAATSASSTQISQTNTGSQPNN
ABJ69412.1      MKINEKQHYKMYKSKGMWVFACLSTCLTVSFFNSGKNVSAATSASSSQISQSNTQTSDAT


YT170           --------------------MADLPSSSDSKSSIGSTISQPTVDKKETSKSDTADNDLT
ABD57319.1      ETTGETAQSSVNSTATASSSSVADLPSSSDSKSSIGSTISQPTVDKKETSKSDTADNDLT
ABJ69412.1       TDESVTQSSSVNGVATEASSKQVALPSSSDSEASSSNTVSQTDDDKNQVASNSTNNTDVT
                                     . *******::* ..*:**.  **::.:...* :.*:*

YT170           KSVTTSDSDKALPTSKTTLPTSNEQVQSSVGQSQTDQSASSATIATN------------A
ABD57319.1      KSVTTSDSDKALPTSKTTLPTSNEQVQSSVGQSQTDQSASSATIATN------------A
ABJ69412.1       KPVTTSDSDKAIINSNTTSPTSDEQAKSSLGQSQTDQSTSSTTIATKATTTVAVDNSAKA
                *.*********:  .*:** ***:**.:**:********:**:****:            *

YT170           VTSDVSQNDQYNEPYRNQYHYSSSQNWINDPNGLFYDSKTGLYNLYYQYNPEGNQWGNMS
ABD57319.1      VTSDVSQNDQYNEPYRNQYHYSSSQNWINDPNGLFYDSKTGLYNLYYQYNPEGNQWGNMS
ABJ69412.1       TTPDISQNDQYDEQYRNQFHYSSNENWINDPNGLFYDSSTGLYNLYYQYNPKGNQWGNMS
                .*.*:******:* ****:****.:*************.************:********

YT170           WGHAVSKDLINWTQEDVAIPMLQNQGWEDFTYTNTTGSLKDKGEVRYVGVPTTNWGDADG
ABD57319.1      WGHAVSKDLINWTQEDVAIPMLQNQGWEDFTYTNTTGSLKDKGEVRYVGVPTTNWGDADG
ABJ69412.1       WGHAVSKDLINWTQEDVAIPMLQNQGREDFTYTNTTGSLKDKGEVRYVGVPTTNWGDADG
                ************************** *********************************

YT170           KKAIFSGSIVVDTNNVSGLGKDAILAFYTADYQIATRKNDGAEDGWGTWIGLTEIQEQHL
ABD57319.1      KKAIFSGSIVVDTNNVSGLGKDAILAFYTADYQIATRKNDGAEDGWGTWIGLTEIQEQHL
ABJ69412.1       KKAIFSGSIVVDTNNVSGLGKDAILAFYTADYQIATRKNDGAEDGWGTWIGLTEIQEQHL
                ************************************************************

YT170           AYSLDGGKTFIQYSKDGNAANPQAIIPTSMNQGGDAANFRDPSVVYDAVNKQYYLTVVSG
ABD57319.1      AYSLDGGKTFIQYSKDGNAANPQAIIPTSMNQGGDAANFRDPSVVYDAVNKQYYLTVVSG
ABJ69412.1      AYSLDGGKTFIQYSKDGNAANPQAIIPTSMNQGGDSANFRDPSVVYDAVNKQYYLTVVSG
                ***********************************:************************

YT170           QQALIYKSSNLLDWTYASKIERENDVGNGVWECPSLVPMKVAGTNETKWVFCISVQQGAH
ABD57319.1      QQALIYKSSNLLDWTYASKIERENDVGNGVWECPSLVPMKVAGTNETKWVFCISVQQGAH
ABJ69412.1       QQALIYKSSNLLDWTYASKIERENDVGNGVWECPSLVPMKVAGTNETKWVFCISVQQGAH
                ************************************************************

YT170           ATGSGMQYYVGNMTADGTWVPESSKTLQNPMTMDSGEDFYAGIPFSNMPDGRTVMLAWQS
ABD57319.1      ATGSGMQYYVGNMTADGTWVPESSKTLQNPMTMDSGEDFYAGIPFSNMPDGRTVMLAWQS
ABJ69412.1      ATGSGMQYYVGNMTADGTWVPESSKTLQNPMTMDSGEDFYAGIPFSNMPDGRTVMLAWES
                **********************************************************:*

YT170           NWSYVDEAKTSPWSGNMTLPRELSLKKNADTTDGYLLTNTVVKEIANNEEANVINKAESN
ABD57319.1      NWSYVDEAKTSPWSGNMTLPRELSLKKNADTTDGYLLTNTVVKEIANNEEANVINKAESN
ABJ69412.1       NWSYTGEANTTPWYGNMTLPRELTLEQNADTTDGYLLANTVIKEIANNEEANVIDQKNST
                ****..**:*:** *********:*::**********:***:*************:: :*.

YT170           FTVSRSDEQVQYEGKQYKISATFSWDEADKPKSVGFKLRVSDDQKYDMIVGYDLTTGLLY
ABD57319.1      FTVSRSDEQVQYEGKQYKISATFSWDEADKPKSVGFKLRVSDDQKYDMIVGYDLTTGLLY
ABJ69412.1       FSISSTDQKVQYDGKQYKISATFSWDESNKPSSVGFKLRVSDDKEYDMLVGYDLTTGLFF
                *::* :*::***:**************::**.***********::***:*********::

YT170           VQRLNTGEPNMGAPRDKMNATVNADGSITITVYVDETSIEAFANDGEKSITQNFFMRPEN
ABD57319.1      VQRLNTGEPNMGAPRDKMNATVNADGSITITVYVDETSIEAFANDGEKSITQNFFMRPEN
ABJ69412.1       VQRLNTGEPNMGAPRDKMNATVNADGSITITVYVDETSIEAFANNGEKSITQNFFMRPEN
                ********************************************:***************

YT170           IGDQATTGVYVYSNDGTTKISDLTINPITSIWNSTGQLTEKFVDENGNTIASDKIQTGRV
ABD57319.1      IGDQATTGVYVYSNDGTTKISDLTINPITSIWNSTGQLTEKFVDENGNTIASDKIQTGRV
ABJ69412.1       IGDQATNGVYVYSDNGTTNISNLTINPIASIWNSVGKLTEKFVDESGNSIAADVVSSGNI
                ******.******::***:**:******:*****.*:********.**:**:* :.:*.:
```

```
YT170          GQSYTSESATIPGYVFVKENTDHINSNQLYTTQNQTITYTYRASQASVVTKDTTLAAGPS
ABD57319.1     GQSYTSESATIPGYVFVKENTDHINSNQLYTTQNQTITYTYRASQASVVTKDTTLAAGPS
ABJ69412.1      GDPYSTTQKDIAGYTFKEVQGSPKGS---FTAQDQTVTYVY--------TKN-PVAGG--
               *:.*:: .  *.**.* : : .  .*   :*:*:**:**.*       **: .:*.*

YT170          AAWNAADNLVGATDADGNALAVSDLTVNGAVDPKTPGTYTVTYSYTDATGNKISKKATVT
ABD57319.1     AAWNAADNLVGATDADGNALAVSDLTVNGAVDPKTPGTYTVTYSYTDATGNKISKKATVT
ABJ69412.1     --------------------------------------NVTAKYVDESGNSIAT-----
                                                      .** .*.* :**.*:.

YT170          VIASKADIVTKDTTMVAGASTIWNAADNFVEAKNADGNALTVSDLMINGTVDSKTPGTYT
ABD57319.1     VIASKADIVTKDTTMVAGASTIWNAADNFVEAKNADGNALTVSDLMINGTVDSKTPGTYT
ABJ69412.1      ------DVVSSGNISDPYSTTQKNIAG-------------------------------
                    *:*:...    . ::*  * *.

YT170          VTYSYTDAAGNKINKEAIVTVIASKADIVTKDTTMVAGPSAAWNAVDNFVEATGADGNAL
ABD57319.1     VTYSYTDAAGNKINKEAIVTVIASKADIVTKDTTMVAGPSAAWNAVDNFVEATGADGNAL
ABJ69412.1      --YTFKEVQG------------------------------------------------
                 *::.:. *

YT170          ALSDLTVNGAVDPKTPGTYTVTYSYTDPAGNKISKEATVTVIASKADIVTKDTTMVAGPS
ABD57319.1     ALSDLTVNGAVDPKTPGTYTVTYSYTDPAGNKISKEATVTVIASKADIVTKDTTMVAGPS
ABJ69412.1      -------------------------TPTGNFTAQDQTVTYVYTKN-------------
                                          *:**  ::: *** : :*

YT170          ATWNAVDNFVEATGADGNALALSDLTVNGAVDPKTPGTYTVTYSYTDPAGNKISKEAIVT
ABD57319.1     ATWNAVDNFVEATGADGNALALSDLTVNGAVDPKTPGTYTVTYSYTD------------
ABJ69412.1      -----------------------------------P----------------------
                                                   *

YT170          VIASKADIVTKDTTKVAGPSATWNAADNLVIATVAMGNALALSHLTVTGSVDSKTPGTYS
ABD57319.1     ------------------------------------------------------------
ABJ69412.1      ------------------------------------------------------------

YT170          VTYSFTDVAGNKISKEAIVTVIASKADIVTKDTTKVAGPSATWNAADNLVIATDAKGNAL
ABD57319.1     -------VAGNKISKEAIVTVIASKADIVTKDTTKVAGPSATWNAADNLVIATDAKGNAL
ABJ69412.1      -------VAGGKVTGD------------------------------------------
                      ***.*:: :

YT170          ALSNLTVTGSVDSKTPGTYTVPYSYTDAAGTKISKEAIVTVIASKADIVTKDTTMVAGPS
ABD57319.1     ALSNLTVTGSVDSKTPGTYTVTYSYTDAAGNKISKEATVTVIASKADIVTKDTTMVAGPS
ABJ69412.1      -------TGSIQSKNP----------SVTGNRSEKPS----------------------
                      ***::**.*          ..:*.: .* :

YT170          AAWNAANNLVSATDADGNALAMSNLTVTGTVDLKTQGTYTVTYTYTDVAGNKISKEATVT
ABD57319.1     AAWNAANNLVSATDADGNALAMSNLTVTGTVDLKTQGTYTVTYTYTDVAGNKISKEATVT
ABJ69412.1     ------------SDQLGNSSQLNNSAKSGLVNTSTS-----------------------
                           :*  **:  :.* : :* *: .*.

YT170          VLTEKETNIEDNTGSSISNDRENPPASITGKGGDDIHQNAKTTMTKKKTETLPQAGNHVN
ABD57319.1     VLTEKETNIEDNTGSSISNDRENPPASITGKGGDDIHQNAKTTMTKKKTETLPQAGNHVN
ABJ69412.1     ------------AGSQSNNDFN-----------SISRLPKTGEGKNEKQTISFVG----
                           :**. .** :            .* : .**   *::.:*:. .*

YT170          ELAIVLGQMILAICVGGILWLKRRVKRV-------------------------------
ABD57319.1     ELAIVLGQMILAICVGGILWLKRRVKRV-------------------------------
ABJ69412.1     VLLVIVGNLLGLIGIK-----KHRSSL--------------------------------
                * :::*:::  * :      *:* .
```

Supplementary Fig. S4. Alignment of the amino acid sequence of putative levanase targeting at degrading long-chain inulin from *L. paracasei* YT170 and related enzymes in the GH32 family showing the highly conserved structure (Gene bank nos. ABD57319.1 in *L. paracasei* 1195 and ABJ69412.1 in *L. casei* ATCC 334)

```
CLUSTAL X (1.83) multiple sequence alignment


CCC77729.1_sucrose-6-phosphate      ---MIWNRKTRYTPYEQWPATKLPQLVAQVRQSKWRMQHHIQPTSGLLND
YP_003923471.1_beta-fructofura      ---MIWNRKTRYTPYEQWPATKLPQLVAQARQSKWRMQHHIQPTSGLLND
YT170                               MKEATWSTAARYQPYSSWAPDYIMKLKAQVAASKWRTKTHVQPDTGLIND
                                         *.  :** **..*..  : :* **.  **** : *:** :**:**

CCC77729.1_sucrose-6-phosphate      PNGFSYFDGQWHLFYQVFPFGPVHGLKSWQHVTSKNLVDWHDEGLAIRPD
YP_003923471.1_beta-fructofura      PNGFSYFDGQWHLFYQVFPFGPVHGLKSWQHVTSKNLVDWHDEGLAIRPD
YT170                               PCSLNFFNNKWHLYYQQFPFGPVHGLKSWAHAVSKDLFNWRRVPGDLLPD
                                    * .:.:*:.:***:** ************ *..**:*.:*:     : **

CCC77729.1_sucrose-6-phosphate      TPYDSHGAYTGTALPIDDQLFIMYTGNVRTADWQRESYQLGAWMDTDNHI
YP_003923471.1_beta-fructofura      TPYDSHGAYTGTALPIDDQLFIMYTGNVRTADWQRESYQLGAWMDTDNHI
YT170                               NEYDSHGAYTGSALVTHGTLRLMYTGNARDDQWHRHSTQLGAVLGADGRL
                                    . *********:**  .. * :*****.*  :*:*.* **** :.:*.::

CCC77729.1_sucrose-6-phosphate      KKLSRPLIAHAPAGYTSSFRDPDLIRTDHGYYALIGAQTTTEIGAILVYF
YP_003923471.1_beta-fructofura      KKLSRPLIAHAPAGYTSSFRDPDLIRTDHGYYALIGAQTTTEIGAILVYF
YT170                               FKDPKPLILTPPTGYTQEFRDPFLFNYEGQTYVLIGGQRPDHTGAILLYA
                                     * .:***  .*:***..**** *:. :   *.***.* . . ****:*

CCC77729.1_sucrose-6-phosphate      SKDLTTWTCQGELNVPANARGYMIECPNLVWIDQQPVLLFCPQGLSQTTT
YP_003923471.1_beta-fructofura      SKDLTTWTCQGELNVPANARGYMIECPNLVWIDQQPVLLFCPQGLSQTTT
YT170                               KQTDKSWRFVAPLSIPDEFCGYMVECPNITFINGKVVLVYCPQGLDQDFF
                                    .:  .:*   . *.:* :  ***:****:.:*: : **::*****.*

CCC77729.1_sucrose-6-phosphate      PYQNIYPNMYLVADQLDLAQAQFTEPHALTQLDDGFDVYATQAINAPDGR
YP_003923471.1_beta-fructofura      PYQNIYPNMYLVADQLDLAQAQFTEPHALTQLDDGFDVYATQAINAPDGR
YT170                               EYENVYPNIALVADSFDPATGNLTH-QRLQNIDKGFDFYATRLANTDDDG
                                     *:*:***: ****.:* * .::*. : * ::*.***.***:  *: *.

CCC77729.1_sucrose-6-phosphate      ALAVSWIGLPEISYPTDRENWAHCLSLVKELTLKDGHLYQNPVAAVDDLR
YP_003923471.1_beta-fructofura      ALAVSWIGLPEISYPTDRENWAHCLSLVKELTLKDGHLYQNPVAAVDDLR
YT170                               TLAISWLGLPDTTYPTDDDGWAGVLSYVRQLTLRDDHVCLYPHPAIKSLR
                                    :**:**:***: :**** :.**  ** *::***:*.*:   * .*:..**

CCC77729.1_sucrose-6-phosphate      -TTAHDLVFEQQR---ATVAALNGSFELLLTVPADKTVTVNIADQQESGQ
YP_003923471.1_beta-fructofura      -TTAHDLVFEQQR---ATVAALNGSFELLLTVPADKTVTVNIADQQESGQ
YT170                               ETAVEDLPVIQQHDDEWTVTNLEGAFELAFTLAAGQKTTIHLPDG-DHDQ
                                     *:..** . **:    **: *:*:*** :*:.*.:..*:::.*  : .*

CCC77729.1_sucrose-6-phosphate      LQVTVDANHGQVMIDRRHTGNSFAEDYGQTRQVELTAHKTIKIRLIIDVS
YP_003923471.1_beta-fructofura      LQVTVDANHGQVMIDRRHTGNSFAEDYGQTRQVELTAHKTIKIRLIIDVS
YT170                               LLIHLDSDSGQGMIQRENRNNG-----GSLRQFGFPAGKTVEIRLFIDVS
                                    * : :*:: ** **:*.: .*.     *. **. :.* **::***:****

CCC77729.1_sucrose-6-phosphate      VFECYIDNGYSVMTGRFFLNATPSRLNVQGDTTAVTGKVWEWRQSEHTGV
YP_003923471.1_beta-fructofura      VFECYIDNGYSVMTGRFFLNATPSRLNVQGDTTAVTGKVWEWRQSEHTGV
YT170                               VFELFIDQGYRVVSGRFFGNEAPTAARITPPSAASDVVSWNLKK-DNGGL
                                    *** :**:** *::**** * :*:  .:   ::*     *: :: :: *:

CCC77729.1_sucrose-6-phosphate      DNNETKIK-
YP_003923471.1_beta-fructofura      DNNETKIK-
YT170                               ---------
```

Supplementary Fig. S5. Alignment of the amino acid sequence of putative sucrose-6-phosphate hydrolase targeting at degrading scFOS from *L. paracasei* YT170 and related enzymes in the GH32 family showing the highly conserved structure (Gene bank nos. CCC77729.1 in *Lactobacillus plantarum* WCFS1 and YP_003923471.1 in *Lactobacillus plantarum subsp. plantarum* ST-III)
